# Ultrasound-Guided Nerve Blocks – Is Documentation and Education Feasible Using Only Text and Pictures?

**DOI:** 10.1371/journal.pone.0086966

**Published:** 2014-02-12

**Authors:** Bjarne Skjødt Worm, Mette Krag, Kenneth Jensen

**Affiliations:** Department of Anaesthesia and Intensive Care, Copenhagen University Hospital Bispebjerg, Copenhagen, Denmark; Friedrich-Alexander University Erlangen, Germany

## Abstract

**Purpose:**

With the advancement of ultrasound-guidance for peripheral nerve blocks, still pictures from representative ultrasonograms are increasingly used for clinical procedure documentation of the procedure and for educational purposes in textbook materials. However, little is actually known about the clinical and educational usefulness of these still pictures, in particular how well nerve structures can be identified compared to real-time ultrasound examination. We aimed to quantify gross visibility or ultrastructure using still picture sonograms compared to real time ultrasound for trainees and experts, for large or small nerves, and discuss the clinical or educational relevance of these findings.

**Materials and Methods:**

We undertook a clinical study to quantify the maximal gross visibility or ultrastructure of seven peripheral nerves identified by either real time ultrasound (clinical cohort, n = 635) or by still picture ultrasonograms (clinical cohort, n = 112). In addition, we undertook a study on test subjects (n = 4) to quantify interobserver variations and potential bias among expert and trainee observers.

**Results:**

When comparing real time ultrasound and interpretation of still picture sonograms, gross identification of large nerves was reduced by 15% and 40% by expert and trainee observers, respectively, while gross identification of small nerves was reduced by 29% and 66%. Identification of within-nerve ultrastructure was even less. For all nerve sizes, trainees were unable to identify any anatomical structure in 24 to 34%, while experts were unable to identify anything in 9 to 10%.

**Conclusion:**

Exhaustive ultrasonography experience and real time ultrasound measurements seem to be keystones in obtaining optimal nerve identification. In contrast the use of still pictures appears to be insufficient for documentation as well as educational purposes. Alternatives such as video clips or enhanced picture technology are encouraged instead of still pictures extracted from basic ultrasonograms.

## Introduction

With the increasing use of ultrasound (US) for various clinical purposes, we are now seeing an immense increase in the use of peripheral nerve blocks for surgical anaesthesia and postoperative analgesia. There is something intuitively pleasing by the fact that you can actually see for yourself - largely without the use of adjuvant, technical surrogate measures - the impact on the structures you are targeting and the extent of local anaesthetics in real time when administering peripheral nerve blocks (PNBs). Studies are forthcoming that document an increased ability to learn the trade of administering PNBs [1], [2] as compared to nerve stimulation techniques. Studies using stimulation thresholds or block success rates as surrogate parameters for proximity of needle point to neural structures have laid the foundation of our current understanding of PNBs, but these surrogate measures may now be considered somewhat redundant [3]. Using ultrasound guidance, we may better visualize the borders of the neural structure itself, anatomical variations, spread of local anesthetic, and potentially hazardous intraneural or intravascular deposits of local anesthetic [4]–[6].

In the wake of these visual advancements, a growing number of textbooks and internet learning tools use ultrasonographic still pictures to visualize neural structures or document their existence in relation to needle approaches. Often, these pictures are processed using graphic overlays that amplify the structure in question, highlighting nerve sheaths, adjacent vessels, fascia, muscles or bony landmarks. These image optimizations are necessary since the structures do not present themselves in a clear and unequivocal manner. Consequently, such textbook presentations are often entirely dissimilar to the representations in clinical life in which variations in neuroanatomy, body composition or inadequate probe placement may render anatomical relationships hard to recognize. A strong selection bias in the choice of representative images must be anticipated in many, if not all, image histories on paper. Finally, image quality may be reduced by inadequate exposure, scan frequency, probe type, focus or hand stability. Finally, hands-on, real-time ultrasonographic investigation has a number of informational tools available that still pictures do not: Relevant probe movement along the long axis of target tissue, angulations to better identify anisotropic structures, rotations for better cross-sectional or longitudinal identifications of target tissue, and repositionings on skin and tissue to separate immobile fascia from mobile nerve structures. These caveats apply to all ultrasound image documentation and must in some respect influence the clinical usefulness and quality of still picture documentation.

We set out to investigate if still picture sonograms are useful for identifying neural structures when doing ultrasound guided (USG) PNBs. When one is learning procedural skills in US, the traditional method is based on a thorough literature study and a close look at still pictures. At least three elements are important in this regard: *1) knowledge of 3D anatomy; 2) recognition of target tissue; 3) procedural skills/the scanning procedure itself*. With minor variations, these elements are all necessary in order to master US [7]. This study will look into how and what is in fact recognized by anaesthesiologists performing US. When defining competence in ultrasonography, the EFSUMB criteria [8] are the natural choice of use. We hypothesize that experts are better at identifying neural structures than inexperienced trainees, and that still picture sonograms are inferior to real time ultrasound when identifying neural structures, but it is yet undetermined if the extent of these differences is clinically relevant.

### Purpose of the study

The purpose of the current study is to quantify – for different competence levels and nerve sizes – the ability to:

- identify nerve gross visibility or ultrastructure using still picture sonograms compared to real time ultrasound, and
- quantify potential gaps in such identification and discuss their clinical or educational relevance.

## Materials and Methods

Consecutive patients undergoing orthopaedic outpatient surgery with the placement of a preoperative USG PNB during a 12-month period (from April 2011 to Otober 2012) at a major university hospital (Copenhagen University Hospital Bispebjerg) were included in the study [9]. All blocks were considered basic nerve blocks of easy-to-moderate complexity. All blocks were in standard use in the department, for either intraoperative anaesthesia or postoperative analgesia, and a dedicated US procedure room was used for all patients. Blocks requiring particular expertise were excluded from the study. US procedures were carried out with the following equipments: SonoSite S-ICU, SonoSite M-Turbo, and BK Medical Flex Focus 400. A linear 5–10 MHz ultrasound probe was used. The target nerve was identified in short axis view. The probe was placed on the following areas: inguinal crease (femoral nerve), parasagittal plane at junction between pectoral and deltoid muscles (infraclavicular nerve), medial and caudal to anterior iliac spine (lateral cutaneous femoral nerve), medial part of inguinal crease (obturator nerve), popliteal fossa above knee-joints (sciatic nerve), midfemoral at sartorius muscle (saphenous nerve) and midneck (interscalene). These areas are those generally used for ultrasound guided nerve blocks (www.usra.ca).

The anaesthetists in our department were divided into four categories of competence in ultrasonography according to the EFSUMB criteria [8]: 1) *novices* with no formal training, 2) *trainees* having completed an USG PNB school but with limited practical experience, 3) *practitioners* with more extensive experience in USG PNBs, and 4) *experts* with extensive experience doing research and quality improvement. All nerve blocks were administered by either trainees or experts in a random fashion, while nerve blocks administered by novices were excluded. No practitioners were employed in the department at the time of the study. Each USG block has been described in detail elsewhere (www.nysora.com; www.usra.ca). Patient demographics (height, weight, age, gender) were recorded on the day of the procedure and the “Vienna scale” designed to classify neural visibility [10] was registered by the observer during all procedures. This scale was introduced by Marhofer in 2008 and classifies the ultrasonographic visibility of neural structures according to the supposed underlying histology or socalled “ultrastructure”: Vienna scale 1) within-nerve structures identifiable; Vienna scale 2) only sheath/epineurium identifiable; Vienna scale 3) no nerve, only surrounding tissue identifiable; or Vienna scale 4) no tissue components identifiable at all. These classifications may be further reduced into even simpler classifications of “gross visibility”: 1) within-nerve structures- or epineurium identifiable), or 2) no tissue components identifiable, or only surrounding tissue identifiable. Two types of observation techniques are used: 1) a real time clinical study using dynamic US, and 2) an armchair study using still picture sonograms of the best possible presentation of targeted neural structures. Our overall study consists of three substudies, as described below.

Nerves were classified according to size in the following manner. Large nerves: intrascalene, femoral nerve, sciatic nerve. Small nerves: saphenous nerve, obturator nerve, lateral cutaneous femoral nerve, infraclavicular nerve.

### Substudy #1 using dynamic ultrasonography on clinical subjects

A prospective cohort of 635 unselected, consecutive patients undergoing USG PNBs were classified according to the Vienna scale, with equipment and US targets as described above. A total of 620 nerves with information about block administrator were recorded (femoral nerve 78, interscalene 232, sciatic nerve 79, saphenous nerve 100, obturator nerve 101, lateral cutaneous femoral nerve 27, infraclavicular nerve 3). 24 clinicians (22 trainees and 2 experts) were asked to classify the ultrasonography of each neural target using dynamic US with a short axis view. The ultrasonographic appearance of the nerve was related to competence level and nerve size.

### Substudy #2 using ultrasonographic still pictures on clinical subjects

**Figure 1:**
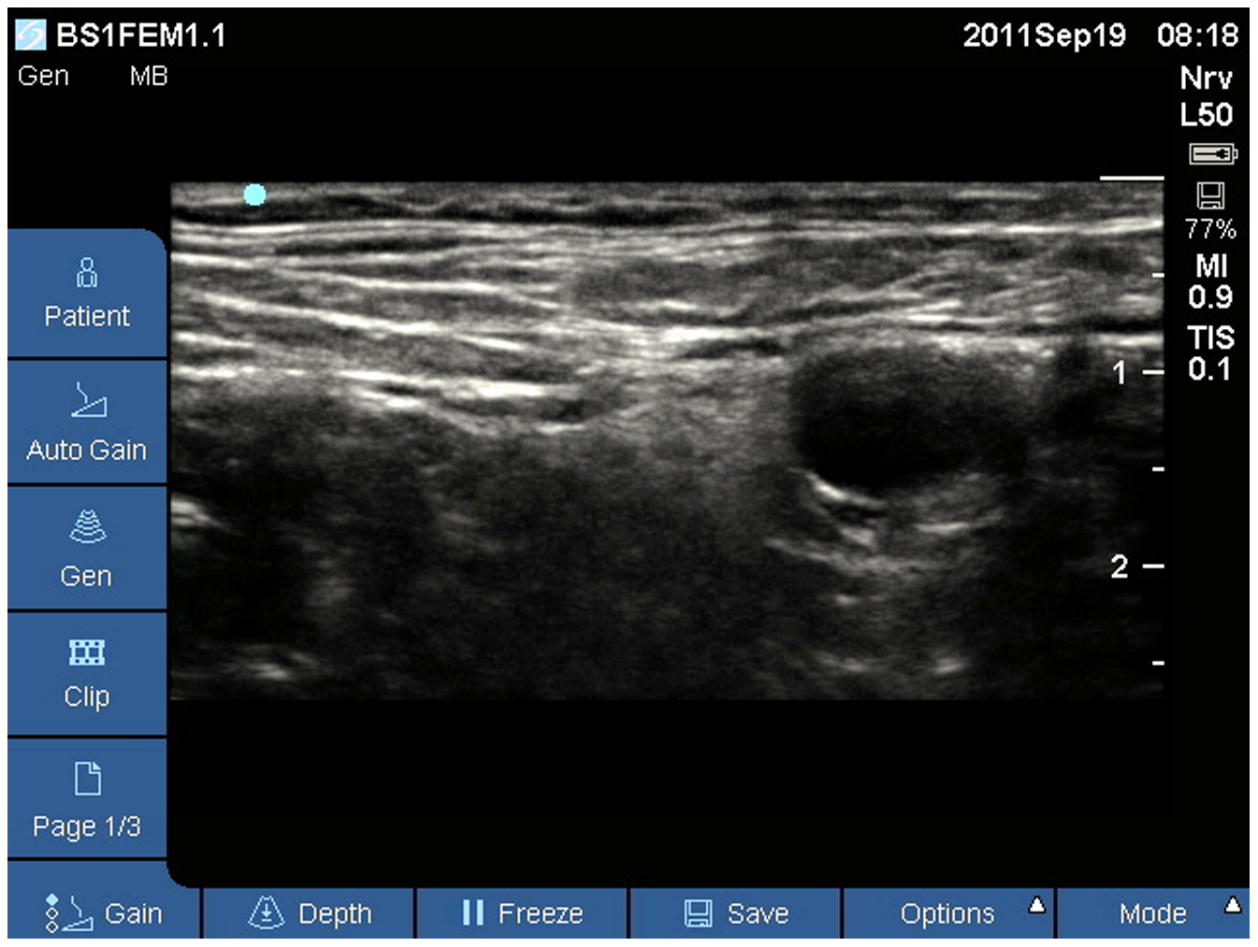
US picture showing a femoral nerve used in substudy #2. This ultrasound image is an example where gross identification of a large nerve was reduced by both expert and trainee observers.

The nerves targeted in substudy #1 were presented as still pictures as demonstrated with figure 1. A random selection of these US images—evaluated during real time ultrasonography to give the optimal representation of the structures—were reevaluated independently by different observers in an internet based solution using a Moodle open-source community tool for learning, and the answers were obtained electronically (www.medviden.dk). Participants were informed of the anatomic location of the ultrasonogram and the nerve in question. A total of 112 ultrasonographic still pictures were reevaluated by 2 experts and 12 trainees. Evaluations were blinded between observers. Nerves identified were the femoral [n = 18], infraclavicular [n = 20], lateral femoral cutaneous [n = 11], obturator [n = 18], sciatic[popliteal [n = 16], saphenous [n = 14], and interscalene [n = 15]. Observers were asked to classify the ultrasonography of the neural targets, and the ultrasonographic appearance of the nerves was related to competence level and nerve size. Interobserver variability using free marginal kappa statistics and interobserver agreement were subsequently calculated.

### Substudy #3 using dynamic ultrasonography on test subjects

Two trainees and two experts each performed ultrasonography with a high frequency linear probe on the same four healthy volunteers, identifying eight neural structures on each side: 1) Large nerves (interscalene, femoral, and sciatic at popliteal level); 2) Small nerves (infraclavicular, lateral femoral cutaneous, obturator anterior and posterior branches, and saphenous). Observers each identified neural ultrastructures systematically in a similar manner and were blinded to the findings of the other observers. Free marginal kappa statistics and overall agreement measures were calculated for each observer group and for each nerve size, as in substudy #2. In addition, Bland-Altman plots were created in order to identify potential systematic bias in pattern recognition between two expert observers and two randomly selected trainee observers. For all interobserver calculations, a free marginal kappa statistic less than 0.30 suggests low level of agreement, and a statistic more than 0.70 suggests high level of agreement. For comparisons of ultrastructure or gross visibility, Fisher's exact test is employed.

## Ethics

Clinical studies performed in Danish hospitals are validated by the Danish Data Protection Agency. No personally data (except from basic demographic values) was recorded. The study was purely educational and the Danish National Committee on Health Research Ethics (DNVK), Region Copenhagen was consulted. They concluded that the study did not require ethics approval (h-4-2013-fsp 41).

Substudy #1 is an unselected, prospective cohort of patients scheduled for elective orthopaedic surgery with one or several peripheral nerve blocks. All patients have given informed consent for anonymous use of Vienna scale classifications. No ethics committee approval is required by Danish law since all nerve blocks and information retrieved is part of the standard documentation at the department. Study design is observational and complies with the Helsinki II declaration.

Substudy #2 is a random selection of still picture ultrasonograms acquired during each block procedure above. All patients have given written informed consent for anonymous use without public photo reproductions. No ethics committee approval is required by Danish law since all information retrieved is part of the standard documentation at the department. Study design complies with the Helsinki II declaration.

Substudy #3 is an observational study of the Vienna scale classification on test subjects during real time ultrasonography. No nerve blocks are administered. All test subjects have given written, informed consent for ultrasonography of peripheral nerves without public photo reproductions. Test subjects have received no payment for their services. No ethics committee approval is required by Danish law since all information retrieved is non-interventional. Study design complies with the Helsinki II declaration.

## Results

All datasets were complete. Demographic characteristics for all substudies are shown in table 1. No adverse events were recorded during the nerve block procedures.

**Table 1:** Demographic chracteristics.

		%
Mean age (SD)	40 (15)	
Gender		
	Male	51
	Female	49
Weight	75 (18)	
Height	173 (12)	
BMI	25.1 (4.8)	
ASA		
	1	40
	2	43
	3	17
	4	0

### Substudy #1

**Figure 2:**
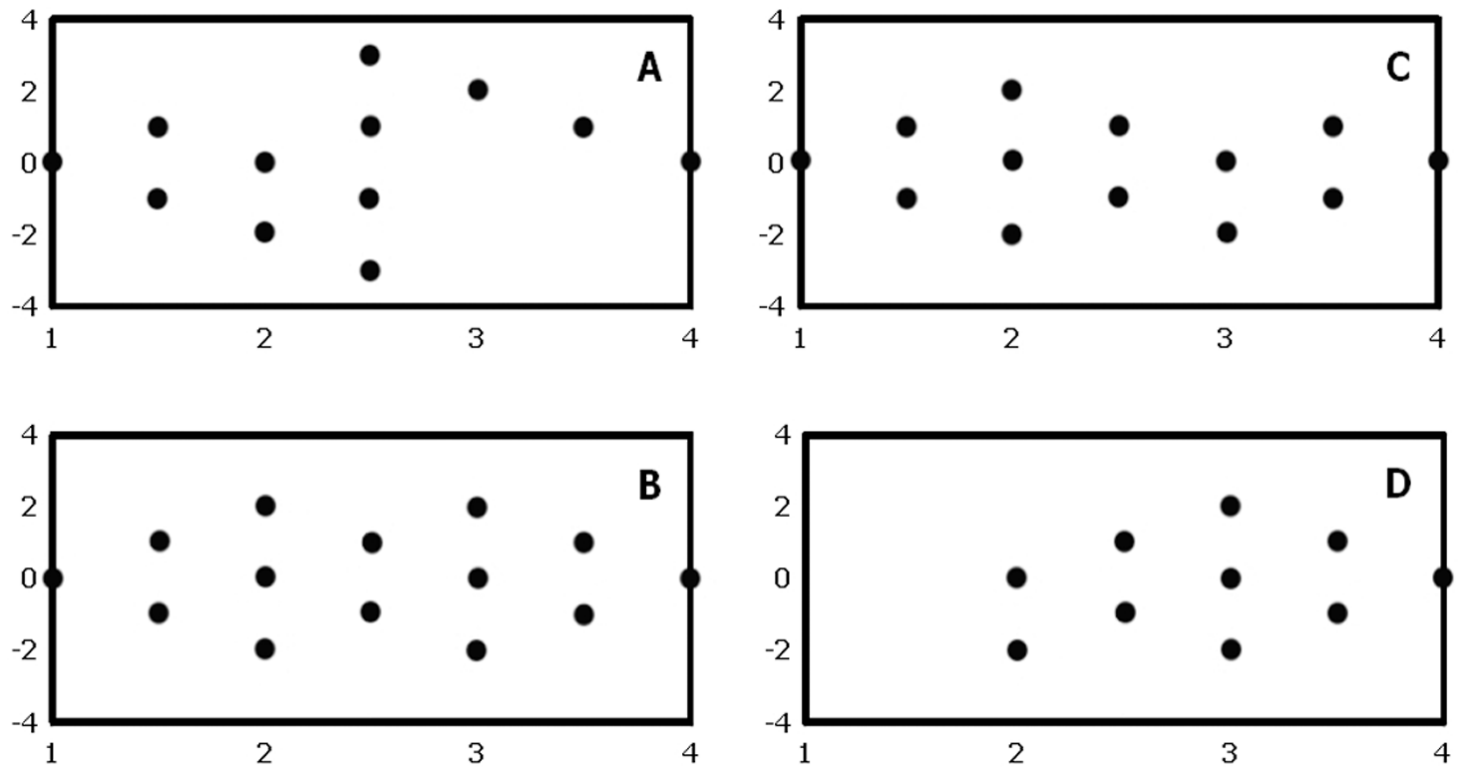
Ultrasonographic visibility of large nerves according to observer competence and method of identification. a, experts; b, trainees. Green, Vienna scale 1; Yellow, Vienna scale 2; Red, Vienna scale 3; Black, Vienna scale 4. #1, substudy 1; #2, substudy 2; #3, substudy 3 (see text for further details).

**Figure 3:**
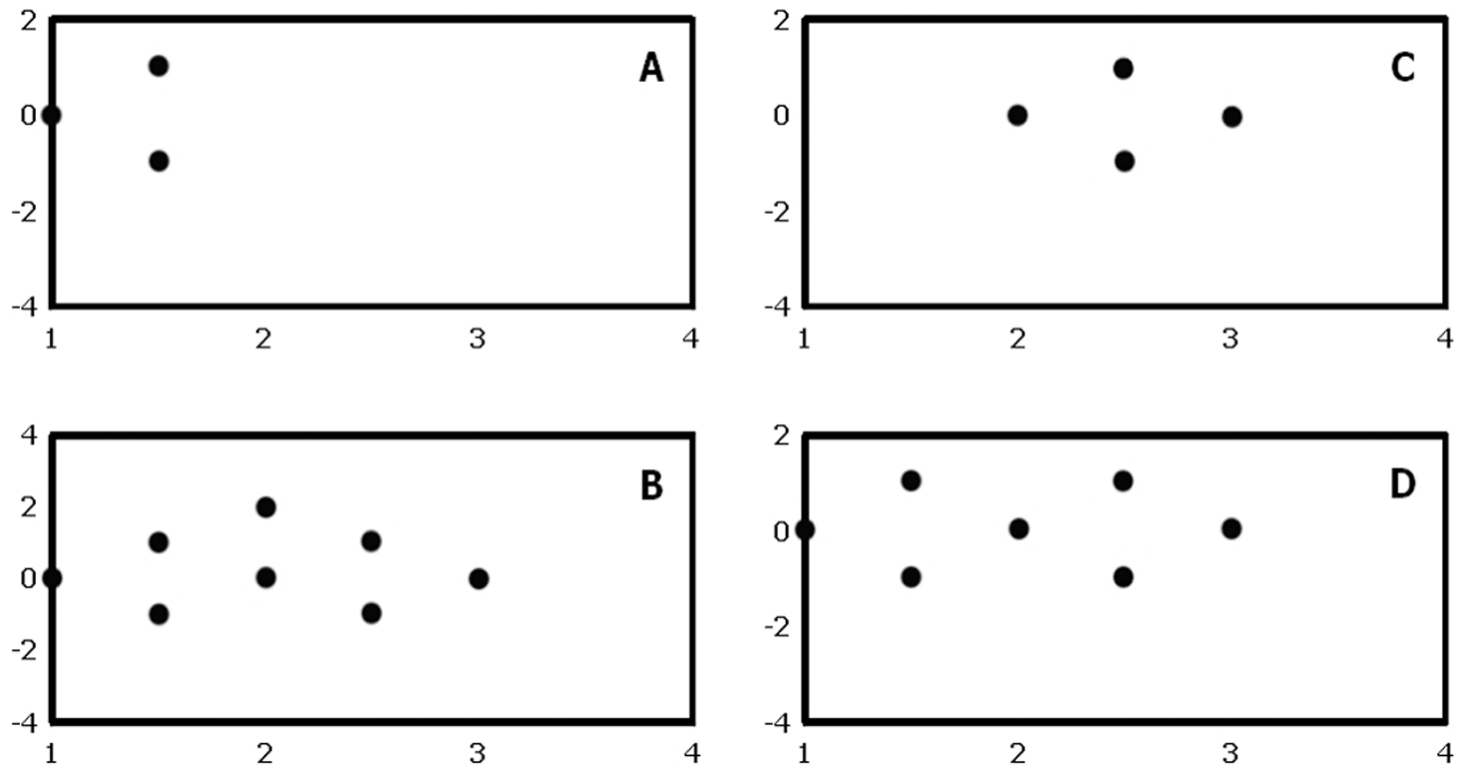
Ultrasonographic visibility of small nerves according to observer competence and method of identification. a, experts; b, trainees. Green, Vienna scale 1; Yellow, Vienna scale 2; Red, Vienna scale 3; Black, Vienna scale 4. #1, substudy 1; #2, substudy 2; #3, substudy 3 (see text for further details).

With optimal examination technique, at least 98% of large nerves in our study are identified by real time US examination regardless of observer competence (Vienna scale 1–2, figure 2; p = 1.00). Trainees are as good as experts in identifying ultrastructure(s) (Vienna scale 1, large nerves: experts 79%, trainees 76%; Vienna scale 1, small nerves: experts 20%, trainees 21%, P = 0.80). About two thirds of small nerves are identified regardless of observer competence (Vienna scale 1–2; experts: 68%, trainees 59%, figure 3; P = 0.14). For both experts and trainees, large nerves are much more easily identified compared to small nerves (p<0.01).

### Substudy #2

By still picture ultrasonography, experts identify 83% of large nerves whereas trainees identify 59% (Vienna scale 1–2, figure 2; p<0.01). Experts identify 42% of small nerves and trainees identify 23% (Vienna scale 1–2, p = 0.03). 68% of the ultrasonograms with small nerve targets cannot be identified (Vienna scale 3–4; experts 58%, trainees 77%, figure 3). For both experts and trainees, large nerves are significantly easier to identify than small nerves (p<0.01). Experts are in good agreement when identifying large nerves, but they only moderately agree when identifying small nerves (Table 2). Trainees largely disagree when studying large or small nerve targets. In general, overall agreements and free marginal kappa values are reduced when evaluating neural ultrastructure compared to gross visibility.

**Table 2:** Overall agreement and free-marginal kappa values for ultrastructure and gross visibility according to observer competence and method of identification.

	Experts	Trainees		
	Ultrastructure	Gross visibility	Ultrastructure	Gross visibility
#2: still pictures, Large Nerves				
Overall agreement	0.49	0.76	0.42	0.66
Free-marginal kappa	0.32	0.51	0.23	0.33
#2: still pictures, Small Nerves				
Overall agreement	0.38	0.55	0.39	0.67
Free-marginal kappa	0.18	0.09	0.18	0.35
#2: dynamic US, Large Nerves				
Overall agreement	0.72	1.00	0.72	0.94
Free-marginal kappa	0.63	1.00	0.63	0.89
#2: dynamic US, small Nerves				
Overall agreement	0.30	0.67	0.80	0.80
Free-marginal kappa	0.07	0.33	0.73	0.60

Legend: For interpretation of these results, see text.

### Substudy #3

Real time ultrasonography (on test subjects) is in accordance with visibility findings of substudy #1 (Figure 2; Chi square-test: p = 1.00). Visible differences between substudy 1 and 3 in terms of Vienna scale prevalence are most probably due to differences in sample sizes.

However, experts identify a percentually larger part of small nerves than trainees (non-signicant differences, 63% vs 40%, p = 0.37). Both experts and trainees highly agree in the identification of large nerves, whereas overall agreement in identifying small nerves is considerably less (Table 2). For experts, overall agreement in identifying ultrastructure is moderate-to-high for large nerves but low to moderate for small nerves. For trainees, overall agreement in identifying both ultrastructure and visibility remains moderate to high.

### Comparisons between substudies

**Figure 4:**
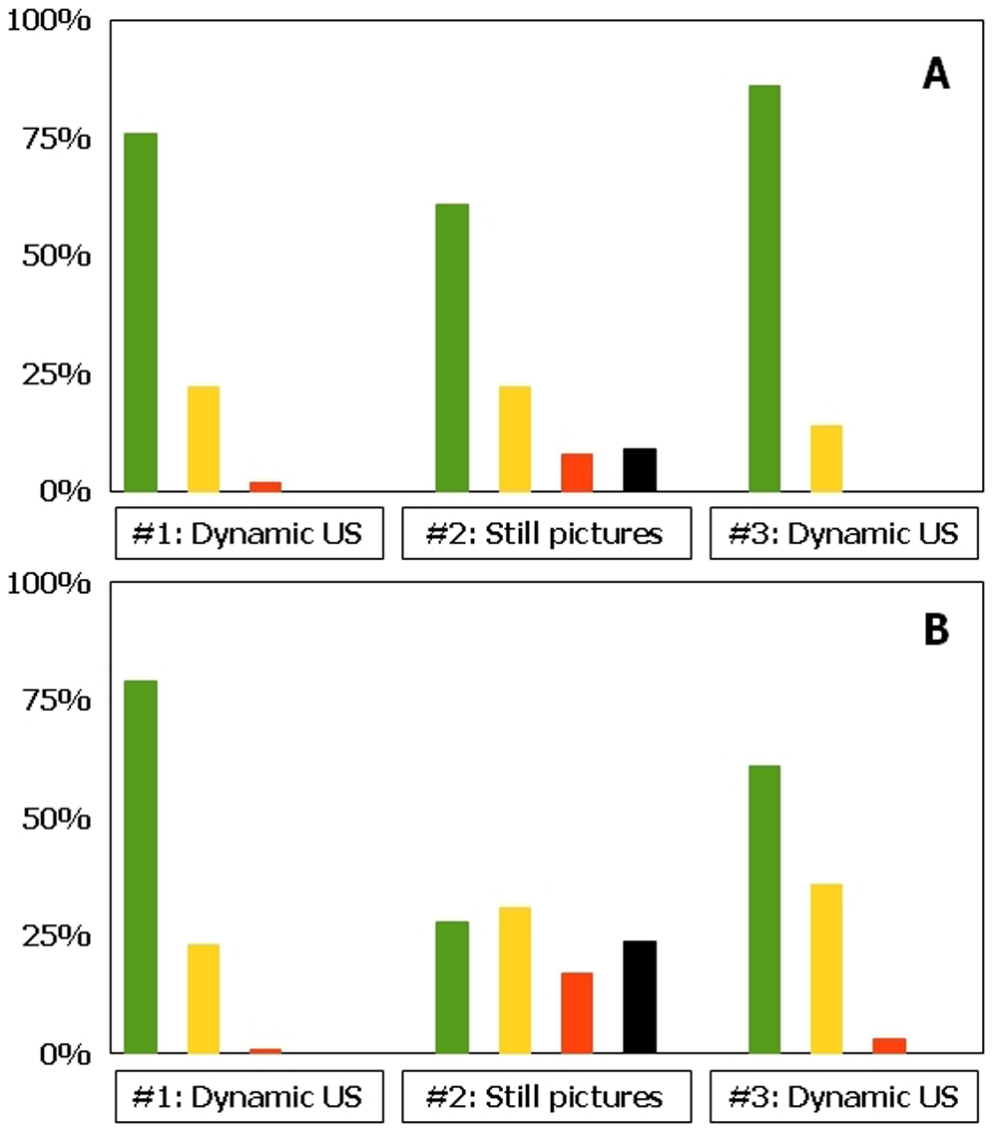
Bland-Altman plots of ultrastructural visibility by still picture sonograms according to observer competence and nerve size. a, experts and large nerves; b, experts and small nerves; c, trainees and large nerves; d trainees and small nerves. X-axis employs mean values of the Vienna scale, while the Y-axis employs observer differences of the Vienna scale. For interpretation of these results, see text.

**Figure 5:**
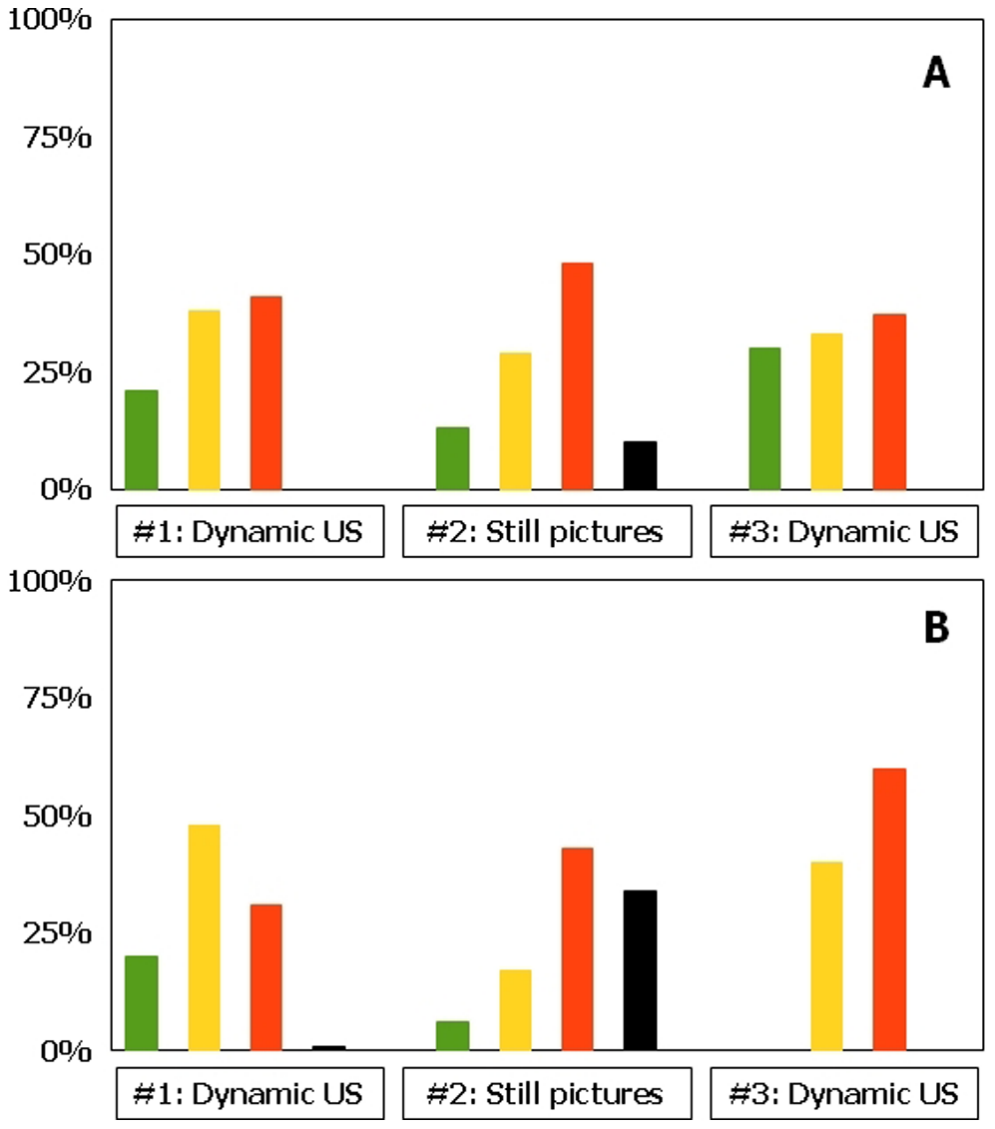
Bland-Altman plots of ultrastructural visibility by dynamic US according to observer competence and nerve size. a, experts and large nerves; b, experts and small nerves; c, trainees and large nerves; d trainees and small nerves. X-axis employs mean values of the Vienna scale, while the Y-axis employs observer differences of the Vienna scale. For interpretation of these results, see text.

Bland-Altman plots suggest that there is no obvious, systematic bias in the assessments between either expert or trainee observers, irrespectively of nerve size or examination method, although agreements are considerably higher in large nerves than small nerves and when using real-time US compared to still pictures (Figure 4–5). When evaluating results from substudies #1 and #2 as a whole, still picture identification is inferior to real time ultrasonography for both experts and trainees (p<0.02). For small nerves, this difference is also true for trainees (p = 0.02), while we have not been able to show this difference for experts (p = 0.16). Again, differences visible on the figure can be due to stochastic variation because of differences in sample size.

## Discussion

### Identification using still picture sonograms

We find that neural identification using still picture sonograms (Vienna scale 1–2) is significantly inferior to real time US examination for both large and small nerve structures. This finding is intuitively supported by the additional information obtained by dynamic ultrasonography; relationships of neural structures relative to tendons, muscles, fascia, vessels, adipose tissue, identification of anisotropy by tilting of the US probe, long axis views, and tissue manipulation during the examination [11], [12]. Finally, neural dislocation by local anaesthetics and needle movements may also help to visualize the nerve [13]. For gross identification of large nerves, identification using still picture sonograms are reduced by 15% (from 98% to 83%) for expert observers (Figure 2A), and by 40% (from 99% to 59%) for trainee observers (Figure 2B). These differences are even further stressed when evaluating intraneural structures, in which trainees identify ultrastructures on still picture sonograms entirely according to chance, compared to the more correct identifications using real time US (Figure 2B). For small neural structures, the gross identification using still images is reduced by 29% (from 59% to 42%) for expert observers (Figure 3A), and by 66% (from 68% to 23%) for trainee observers (Figure 3B). For both large and small nerves, trainees are unable to identify any anatomical structure at all in 24 to 34% of sonograms (Vienna scale 4; Figures 1B and 2B), compared to similar difficulties among experts in 9 to 10% (Vienna scale 4; Figures 1A and 1B). This is a major failure of still picture sonograms for both groups of observers because such problems should arise in less than 1% of cases during optimal, real time sonography (Figure 2 and 3). Investigation into interobserver variation yields consistently low free marginal kappa values for both groups of observers, never mind the nerve sizes or ultrastructural elements to be identified (Table 2), with the only exception being the experts' study of large nerves that yield moderate kappa values and good overall agreement. In contrast, kappa values are consistently higher during real time ultrasonography, except for the expert interpretation of small nerves (Table 2).

### Clinical relevance

Historically, US measurements have been found to be observer dependent with a high positive predictive value and a low negative predictive value [14]. In other words, just because we cannot identify the nerves, it does not mean that they are not present at the location. USG PNBs present a similar problem. Our study showed that even for experts, choosing a single (although optimized) still picture sonogram from the PNB procedure for documentation purposes reduced later gross identification of nerves by 15% to 29% (large vs. small nerves). This loss of information is further illustrated as 9 to 10% (large vs. small nerves) of the still picture sonograms are rendered useless since no anatomical structures can be identified at all. While some may find these statistics better than no documentation at all, we find this quality reduction to be considerable from a clinical viewpoint; the use of ultrasonographic documentation rests on the concept of a reproducible, visual proof of the clinical procedure that may become part of a medicolegal dispute or help define the extent and cause of potential nerve damage following the procedure. Quality gaps in such documentation undermine the credibility and clinical applicability, and should be addressed in detail before general use. Our study did not include graphical enhancers or textual description of the still pictures. To our knowledge graphical enhancers cannot be placed on still pictures using the most common ultrasound machines, but if integrated this could be valuable to avoid information loss from dynamic examinations.

In order to administer ultrasound guided nerve blocks, our findings suggest that the identification of large nerves do not require in depth training. For smaller nerves, on the other hand, in depth training is required.

### Educational relevance

The purpose of using still picture sonograms for education is to have the direct visual appearance of the target tissue that the trainee will recognize during the leap from anatomy textbooks to clinical reality [15]. It rings intuitively true that this leap is facilitated by pictorial accounts from clinical life and this is also true in most cases where pictures are shown in order to demonstrate the clinical findings. However, while experts may feel confident that the still pictures presented to trainees in books, presentations or on the internet adequately serve this purpose, our results strongly suggest that half the neural targets will not be identified by the trainees, and that as many as *one in three* will be unable to see anything meaningful from these still picture sonograms if not assisted by graphical enhancers or explanations. A future study investigating if picture optimizations such as arrows, composite drawings, colours etc. might facilitate pattern recognition is needed.

Our findings suggest that trainees have problems identifying small nerves and nerves at still pictures. Maybe the focus should be pattern recognition – not by still pictures but on the dynamic examination, and this is in fact how US anatomy should be taught.

### Conclusion

Our results suggest that documentation for later use and the visual skills responsible for identifying neural structures by ultrasonography *cannot* be facilitated with any consistency by using still picture sonograms.

We don't think the use of still picture sonograms when documenting USG-PNB's is relevant. To much information is lost from the dynamic examination. The information loss could however be minimized using video or graphical enhancers. Some new UD machines are incorporating such features.

The educational implications infer that much more dynamic methods (such as video clips) or enhanced picture methods. should be used in textbook materials, presentations or bedside teaching situations. Limitations aside, we are nevertheless confident that stand-alone still picture sonograms have limited applicability for educational purposes. We suggest that educational material should be optimized beyond the use of still pictures, one possible method being video clips of USG procedures in internet based solutions, either alone or with target structure optimized still pictures.

We propose that further studies be undertaken to clarify the clinical value of potential alternatives such as video clips or even surrogate measures such as extent, quality and duration of the PNBs, even though some critics would find surrogate measures *altmodisch* in this day and age.

However, when even experts cannot tell one structure apart from the next, perhaps we should realize that we are using this US technology for more than it can actually deliver, and temporarily retrace our steps.
